# How Long Are Long Tandem Repeats? A Challenge for Current Methods of Whole-Genome Sequence Assembly: The Case of Satellites in *Caenorhabditis elegans*

**DOI:** 10.3390/genes9100500

**Published:** 2018-10-16

**Authors:** Juan A. Subirana, Xavier Messeguer

**Affiliations:** 1 Department of Computer Science, Universitat Politècnica de Catalunya, Jordi Girona 1-3, 08034 Barcelona, Spain; peypoch@cs.upc.edu; 2 Evolutionary Genomics Group, Research Program on Biomedical Informatics (GRIB)–Hospital del Mar Research Institute (IMIM), Universitat Pompeu Fabra (UPF), Dr. Aiguader 86, 08003 Barcelona, Spain

**Keywords:** satellite DNA, tandem repeat sequences, *Caenorhabditis elegans*, genome sequencing

## Abstract

Repetitive genome regions have been difficult to sequence, mainly because of the comparatively small size of the fragments used in assembly. Satellites or tandem repeats are very abundant in nematodes and offer an excellent playground to evaluate different assembly methods. Here, we compare the structure of satellites found in three different assemblies of the *Caenorhabditis elegans* genome: the original sequence obtained by Sanger sequencing, an assembly based on PacBio technology, and an assembly using Nanopore sequencing reads. In general, satellites were found in equivalent genomic regions, but the new long-read methods (PacBio and Nanopore) tended to result in longer assembled satellites. Important differences exist between the assemblies resulting from the two long-read technologies, such as the sizes of long satellites. Our results also suggest that the lengths of some annotated genes with internal repeats which were assembled using Sanger sequencing are likely to be incorrect.

## 1. Introduction

The newest generation of sequencing platforms, known as long-read or third-generation sequencing platforms, can generate super-long reads which are orders of magnitude larger than reads from Next-Generation Sequencing platforms like Illumina (Illumina Inc., San Diego, CA, USA). For example, Oxford Nanopore’s (Oxford Nanopore Technologies Inc., Oxford, UK) is only limited by the size of the DNA molecules available; thus, reads up to 1 Mb could be obtained. Details of the different sequencing technologies available have been recently reviewed thoroughly [1]. The main drawback of long-read sequencing is the high error rate of the sequences obtained, a problem which is addressed by determining the consensus sequence from multiple overlapping reads; various software tools are available to optimize overlap and obtain the correct sequence [2]. For a whole-genome assembly, the completeness of the assembly depends on both the quality of the reads and the methods used to correct errors. Repetitive genome regions are a major hurdle, regardless of the platform and methods. Satellites or tandem repeats offer an excellent test case to evaluate and compare the different assemblies. Our previous studies on nematode satellites [3,4] and the availability of two high-quality genome assemblies of *Caenorhabditis elegans* [5,6] prompted us to compare their satellites with the classical reference sequence which was obtained over 15 years ago by the Sanger method [7] and has since been considerably improved [8].

Satellites in nematodes have several characteristics which make them the perfect candidates to evaluate the differences between alternative genome assemblies. Firstly, they are very abundant. Secondly, while they are composed of tandem repeats, individual repeats show small differences in size and sequence: they are not uniform [3]. Furthermore, many satellites can be grouped into families with a related sequence, often spread across several chromosomes, which may create problems to map them accurately. Ideally, assemblies from different sequencing platforms should give identical results for the size and internal variability of satellites. If the results do not coincide, it would suggest that some sequencing platforms are capable of producing higher quality assemblies than others.

In summary, we found that short satellites (below 1 kb) are identical in most cases, whereas long satellites (>4 kb) are always much longer in the long-read assemblies. However, the results generated from the two long-read platforms, PacBioand Nanopore, do not always coincide. We conclude that these platforms give more accurate results for long satellites but should be improved for the correct description of the longest satellites (>20 kb). On the other hand, we found few variations in sequence composition from the reference sequence; no new satellites were discovered by the long-read assemblies, which indicates that the new methods provide excellent overall results when properly used.

## 2. Materials and Methods

The new long-read assemblies can be downloaded from the supplementary material of the respective publications. The LH assembly is based on the PacBio technology (Pacific Biosciences of California, Inc., Menlo Park, CA, USA), with a total length of 103.01 Mb distributed over 108 contigs [5]. The VC2010 assembly is based on Nanopore, with a total length of 104.32 Mb, distributed over 60 contigs [6]. In both cases, the assembly was carried out with the help of data obtained from the Illumina platform. The genome lengths of the newer assemblies differ from the standard genome length of 100.29 Mb, version WB235 or ce11. The genome was downloaded from the UCSC website [8].

We determined the location and orientation of contigs in the new long-read based assemblies with respect to the WB235 reference sequence using the Multiple Genome Comparison and Alignment Tool (M-GCAT) method [9,10]. M-GCAT is a multiple genome alignment tool based on the search of maximal unique matches (MUMs) between genomes on both strands. First, a set of anchor MUMs is found: all MUMs shorter than a specific parameter (minimum anchor length) or randomly found (shorter than log base four on the length of the genome) are discarded. This set of anchor MUMs divide the genome in several short parts, which are subsequently subjected to a recursive search of MUMs. This recursive search is repeated until the length of the target sequence is shorter than a given parameter (100 bases, in our case). Finally, neighboring MUMs which are separated by less than a given parameter (in our case, 2000 bases), are grouped into clusters. These clusters determine the regions of similarity between the original genomes, and M-GCAT gives a percentage of cluster coverage. 

In this paper, we describe the similarity we found between the contigs of VC2010 and the chromosomes of WB235 with M-GCAT. Those contigs with a high index of coverage were selected and concatenated at the location in the corresponding genome. This process gave a set of six VC2010 assembled chromosomes that were compared with the chromosomes of WB235, providing us with a complete telomer-to-telomer comparison with a very high coverage index (97%, 98%, 97%, 98%, 97%, and 98% for chromosomes I–V, X). A graphical comparison is presented in Supplementary Figure S1. Of the 60 contigs in the assembly, only 44 could be aligned. The remaining 16 contigs correspond to four short unplaced contigs and to bacterial contamination. These results confirmed the positions reported for the VC2010 contigs (Supplementary Table 2b [8]). A very high coverage index of 99% was also found for all chromosomes in the case of LH, but only 72 contigs (out of 108) could be aligned. The remaining 36 contigs correspond to a mixture of unplaced and contaminating contigs.

The position, length, and average repeat size of satellites in the three assemblies were determined with the SATFIND program, which is available on our website [11], is described in great detail elsewhere [3], and can also be downloaded from Dryad [12]. The program allows a precise definition of satellites (repeat size, number of repeats, and internal regularity), which is not easily obtained with other programs. It allows the user to search for arrays of any short sequence of a prefixed size without internal repetitions and repeated a minimum number of times in regions with a fixed size. In this paper, we have used the SATFIND program to identify satellites formed by at least four repeats in 800 bp regions. Once a satellite is located, the program continues its search along the genome until no further neighboring repeats are detected. In this way, repeats of 10–200 nucleotides repeated at least four times can be positioned in the genome with no upper limit for the number of repeats in the satellite. In order to eliminate the most irregular satellites, we only accepted those with at least 60% of their repeats with an identical length (±1 bp). For the detection of longer satellites (>4 kb), which usually have higher repeat variability, we only required that 10% of them had an identical length. Once the satellites were obtained, satellites in aligned regions of the three assemblies were compared. We found a clear correspondence in the position of all satellites longer than 4 kb in all three assemblies (Supplementary Datafile S1). Note that SATFIND gives a consecutive number for each contig in the assembly: the corresponding fasta code of each contig is given in Supplementary Datafile S1.

We should note that satellites were often found at the end of contigs in both LH and VC2010 assemblies. For example, there are 21 satellites longer than 4 kb at the ends of some VC2010 contigs, which represent 12.1% of the satellites in Supplementary Datafile S1. In such cases, their length is probably not accurate and contributes to the differences in size between some satellites in equivalent genomic regions. An adequate quantification was not possible for the LH assembly, since there are short gaps between several contigs.

## 3. Results

A comparison of satellite lengths shorter than 4 kb between WB235 and VC2010 genome assemblies is presented in Table 1. Both methods detect similar satellites, but only 86.4% of satellites shorter than 1 kb have identical length, whereas identity decreases rapidly above this length. Similar results were found when the LH assembly was used in the comparison, but a detailed comparison is not warranted, since several contigs which contain satellites could not be assembled.

Longer satellites have a higher repeat variability and were analyzed with a different set of SATFIND parameters: we required that only 10% of the repeats had an identical length, as explained in the ‘Methods’ section. We detected 174 satellites in VC2010 with a satellite length over 4 kb; we found a corresponding satellite in WB235 for all of them. In general, all VC2010 satellites are longer than in WB235; only three are shorter, and six are identical in both assemblies. In the case of LH, it was also possible to locate 165 satellites in the same positions. They represent 94.8% of the satellites in the two other assemblies. A detailed comparison is given in Supplementary Datafile S1.

The increase in genome length due to the longest satellites (>4 kb) was also estimated by comparing the total satellite lengths in the WB235 and VC2010 assemblies. In the latter case, the 16 contaminating plus unplaced contigs correspond to 1.42 Mb of the assembly. The total length of satellites over 4 kb is 3,290,520 bases in VC2010 and 773,821 bases in WB235 (Supplementary Datafile S1), which represents a difference of 2.52 Mb due to satellites. These values nearly entirely explain the difference in the genome assembly size between WB235 and VC2010. The genome length of VC2010 excluding them would be 100.38 Mb, practically identical to that of the WB235 assembly (100.29 Mb).

**Figure 1:**
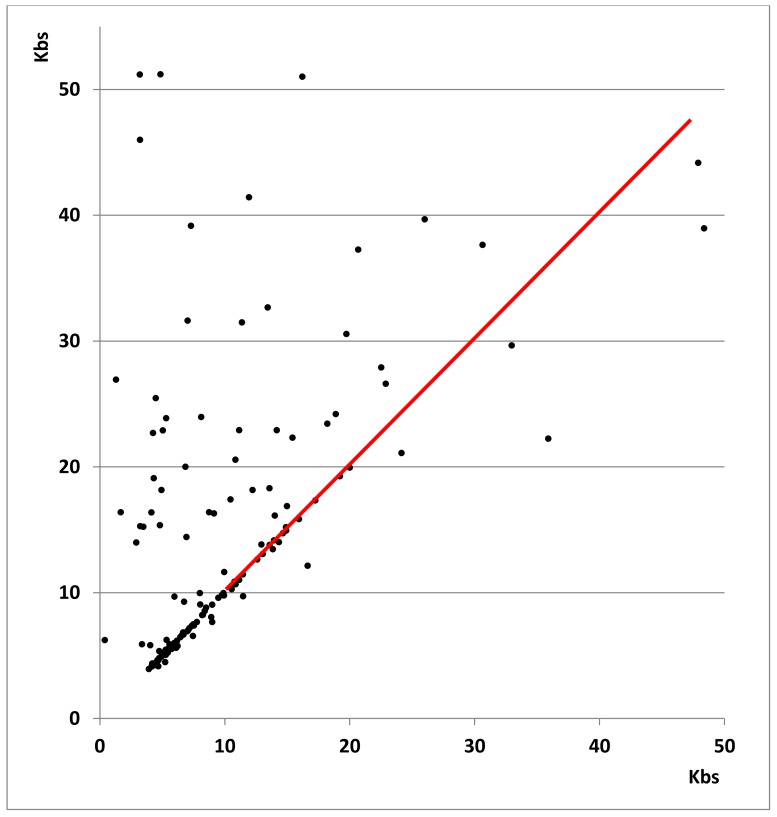
Comparison of satellite lengths in the two new *Caenorhabditis elegans* genome assemblies. Satellites with an identical repeat found in equivalent genomic regions are compared. The length of satellites over 4 kb in the VC2010 assembly is represented as a function of their length in the LH (PacBio) assembly. The red line indicates an identical length of satellites in both assemblies. Only a few satellites in the VC2010 assembly are shorter than in the LH assembly. In the size range 4–20 kb, 68% of the satellites have an identical size. Lengths are different for all 40 satellites over 20 kb; five of them, over 55 kb, are omitted from the figure. Details for all these satellites are given in Supplementary Datafile S1.

**Figure 2:**
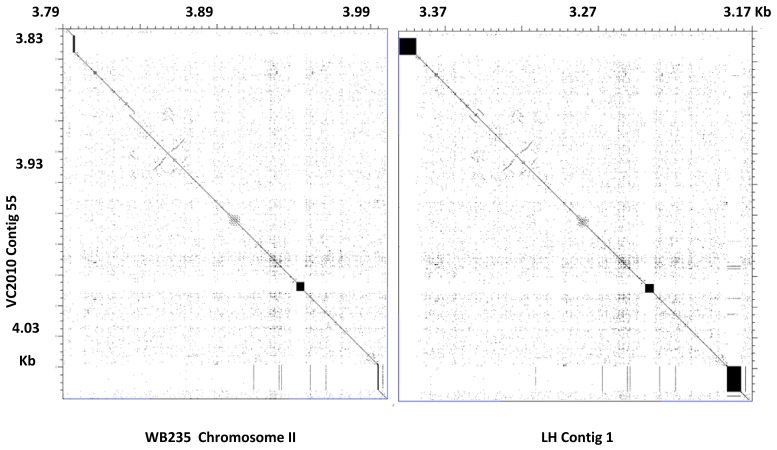
Comparison of a genome region in the three assemblies. The ordinate corresponds to VC2010, and the abscissa to the other assemblies, as indicated in the figure. Three satellites are present in this region, with repeats 24, 79, and 20 bp (from left to right). Their exact coordinates are given in Supplementary Datafile S1. The three satellites are longer in VC2010, especially when compared with WB235. Only the short satellite with repeat 79 is identical in length (5389 bp) in both VC2010 and LH. The neighboring genome regions are identical in LH and VC2010, whereas a small gap and a repeated region are detected in VC2010 and LH at the approximate corresponding position 3,835,000 in WB235. A gene (*oac-4*) is present here [8]; according to the VC2010 and LH data, it should be longer by about 2 kb. The figure was prepared with the Dotter program [13].

**Figure 3:**
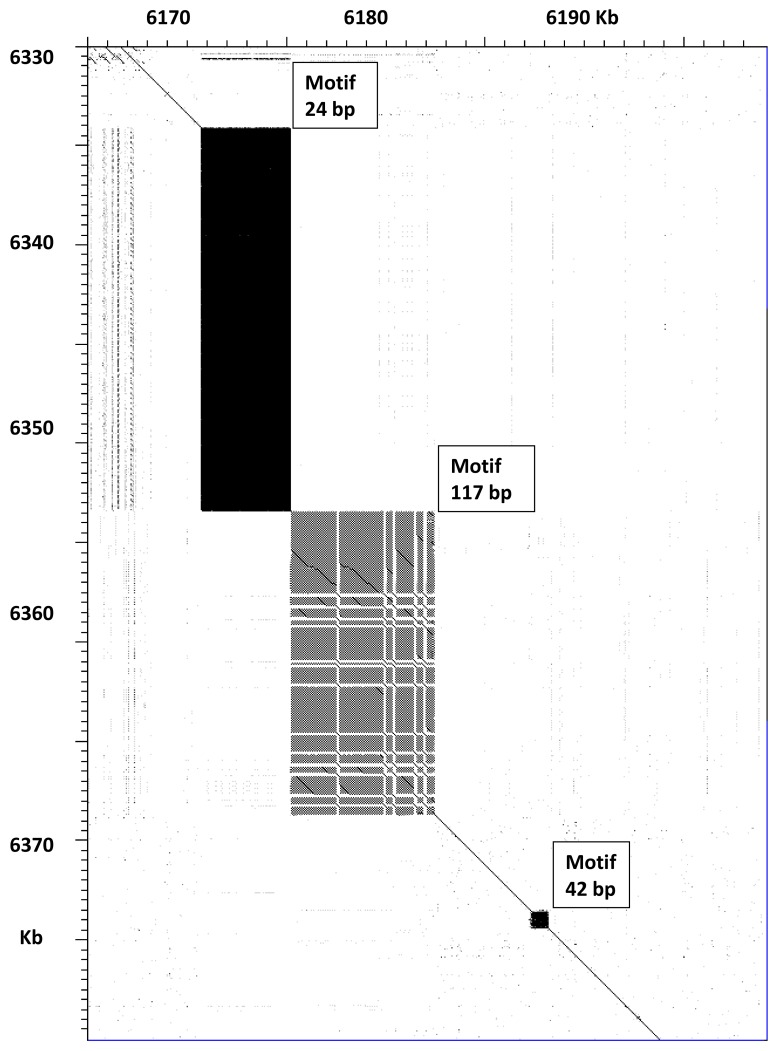
Comparison of satellite lengths in the titin gene of *C. elegans.* Dot plot of part of the titin gene (*ttn-1*), demonstrating its expansion in the Nanopore VC2010 assembly. The figure was prepared with the Dotter program [13]. Coordinates are given in kilobases; WB235, chromosome V, in the abscissa, and VC2010 in the ordinate. This region has three satellites which correspond to amino acid repeats in the protein.

The longer satellites found in the new assemblies suggest that the Sanger-based assembly may produce truncated satellite sequences, but it is also possible that the new assemblies artificially increase the length of some satellites; the availability of the two newer assemblies allowed us to examine each of these possibilities. In the size range 4–20 kb, 68% of the satellites (87/128) have an identical size within 5% (Figure 1 and Supplementary Datafile S1). The lengths determined by the new methods should therefore be considered correct in such cases; thus, the length reported for these satellites in the standard reference assembly is erroneously too short. For satellites over 20 kb in length, there is no agreement; satellites are in general much longer in the Nanopore assembly (VC2010) than in the PacBio assembly (LH). It is not clear which of the two sequencing platforms provides more accurate results: either VC2010 artificially increases the length of some satellites or LH truncates them. Note that the software used in either case for the alignment of reads is also crucial to provide a correct assembly. In any case, the results we obtained (Supplementary Datafile S1) allowed a detailed comparison for any region of interest, as shown in Figure 2 and Figure 3.

We should add that if two satellites are identical in size across two assemblies, this does not guarantee that the sequences of the two satellites are identical throughout their length. Close inspection of the overall sequence showed that differences such as indels or single-base mutations were detected. In the most favorable cases, these differences only represented about 0.1% of the whole satellite.

A striking difference between the three assemblies is the absence of telomere tandem repeats at the end of chromosomes in VC2010, whereas the LH assembly has long telomere sequences (100–550 repeats) in eight chromosome ends. In contrast, the standard ce11 reference assembly has shorter telomere sequences (20–110 repeats) in all chromosomes.

Finally, it should be noted that we detected a few satellites which are part of genes which code for amino acid repeats: they are included in Supplementary Datafile S1. In most cases, the length of these regions coincides in the LH and VC2010 assemblies, which indicates that the values reported in WB235 should be updated. A very clear example is presented in Figure 3 for the *ttn-1* gene. This gene encodes for a titin protein, which contains multiple repeated domains and several regions of tandem short repeat sequences. It may produce several polypeptides of various lengths due to alternative splicing [14]. A comparison between VC2010 and WB235 for part of this gene is shown in Figure 3; this region contains three satellites. The satellite with a 24 bp repeat increases in length from 4465 to 19,240 bp, whereas the satellite with a 117 bp repeat increases from 6979 to 14,926 bp. The smaller satellite with a 42 bp repeat is identical in both cases. The PacBio LH assembly gave very similar results for this genome region, with only 17 single-base indels in the whole 117 bp satellite. Thus, the new long-read assemblies showed that the predicted size of titin protein should be updated by increasing the length by 7574 amino acids. Titin is one of the largest known proteins, reported to be 18,652 amino acids long in WB235 [8], but its actual predicted size should be 26,136 amino acids, if all exons in the gene are expressed.

## 4. Discussion

The main purpose of this paper was to compare the length of satellites in three different assemblies of the *C. elegans* genome. The first step in our analysis was to align the new LH and VC2010 assemblies to the reference WB235 assembly. As shown in Supplementary Figure S1, a high-quality overall alignment was created. However, if the alignment was expanded, numerous small differences appeared, including indels and local inversions of a few kilobases. We have not analyzed these differences, since they do not prevent the comparison of satellites. We found a perfect correlation of satellite positions in the different assemblies, although their length was often different.

We found that long satellites (>4 kb) are longer in the new long-read assemblies. This is not surprising, since the WB235 reference assembly was created using Sanger sequencing, which uses short genome sequences; long arrays of homogeneous repeated sequences cannot be properly aligned in this way. However, only half of the satellites have an identical length in VC2010 and LH. The other half is usually longer in VC2010 than in LH. This result might be attributed to the starting raw sequences used for assembly by the Nanopore method (VC2010), which are longer than in the PacBio method (LH). However, we cannot exclude that the different software used in the assembly may artificially increase the size of the VC2010 satellites, at least in some cases.

Intriguingly, in 50% of the cases, the satellite size is nearly identical in both LH and VC2010 assemblies. This is surprising, as satellites are considered to be highly variable, and these two assemblies have been obtained from different populations of *C. elegans*. It appears that size preservation may be an intrinsic feature of some satellites, with a possible biological significance. This question could be investigated by comparing satellites between many individuals, but unfortunately these data are not available at this time.

In summary, our results show that the overall difference in length of the new long-read genome assemblies with respect to the standard WB235 reference sequence can be mostly attributed to the increased length of satellites. The new assemblies also show that at least 165 satellites in WB235 have to be corrected, since their actual length is significantly longer. However, new methods need to be developed in order to obtain an accurate value for the length of the longest satellites.

## Figures and Tables

**Table 1:** Comparison of satellite lengths in the WB235 (Sanger) and VC2010 (Nanopore) assemblies.

Size Range of WB235 Satellites (kb)	Identical/Total Number	% Identity
<1	1231/1425	86.4
1–4	75/182	41.2
>4	2/65	3.1
Total	1308/1672	78.3

The length of satellites found in corresponding regions of the two assemblies is compared. Satellites are considered identical in the two assemblies when their repeat motif coincides, and the total length of the satellite differs by less than 5%.

## Supplementary Materials

The following are available online at http://www.mdpi.com/2073-4425/9/10/500/s1: Datafile S1: Excel file comparing the properties of satellites found in equivalent genomic regions in the three assemblies; Figure S1: Alignment of the WB235 chromosomes with the VC2010 contigs.
