# Supplementary material for: How Long Are Long Tandem Repeats? A Challenge for Current Methods of Whole-Genome Sequence Assembly: The Case of Satellites in Caenorhabditis elegans
Source: Genes (Basel). 2018 Oct 16;9(10):500. doi: 10.3390/genes9100500 (PMC6210790; doi:10.3390/genes9100500)
Supplement: Supplementary file 1 [file genes-09-00500-s001.zip › Supl_files/Figure_S1+caption.docx]

**Chromosome I**
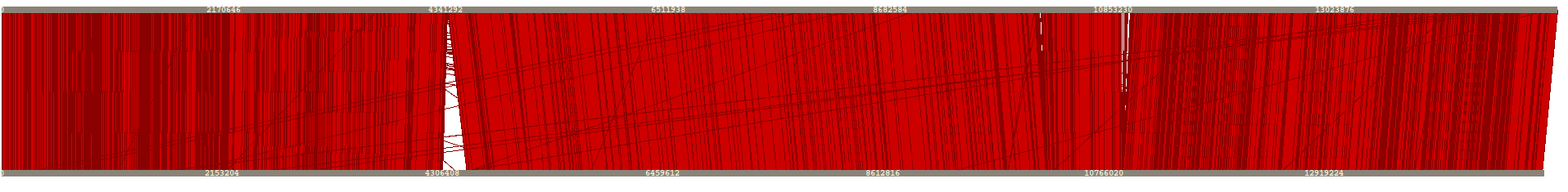


**Chromosome II**
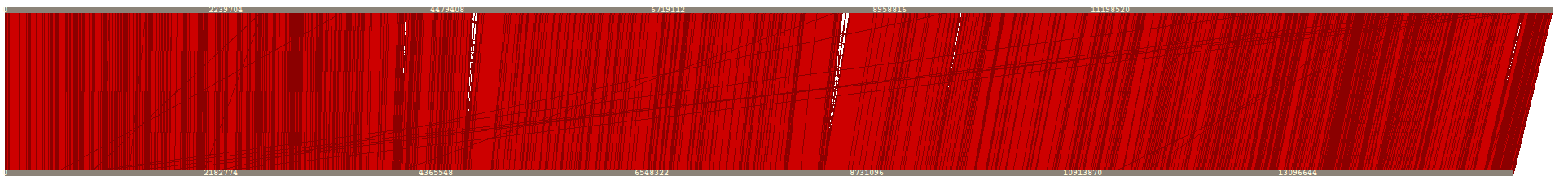


**Chromosome III**
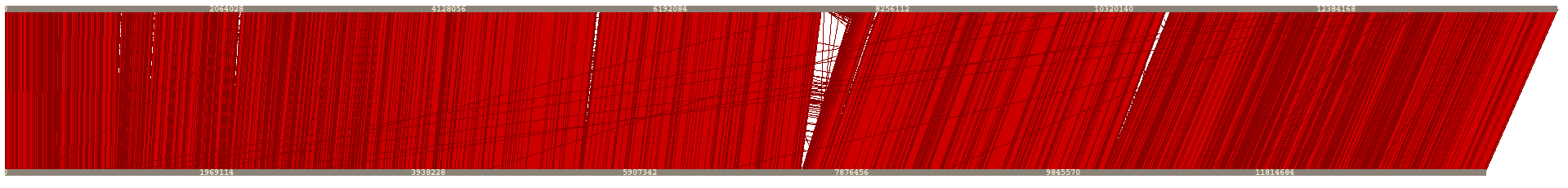


**Chromosome IV**
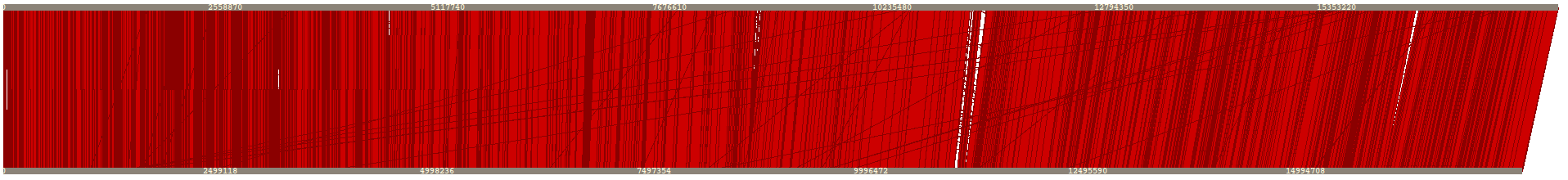


**Chromosome V**
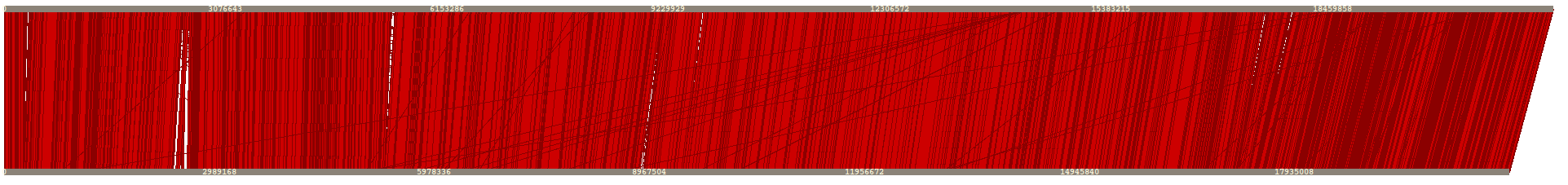


**Chromosome X**
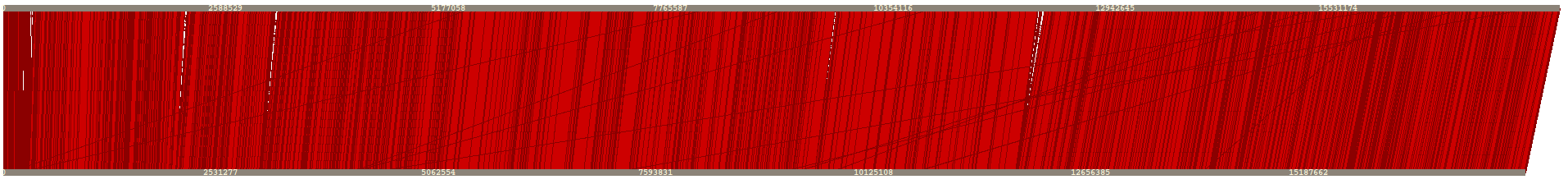


**Figure S1**. Correspondence of the aligned contigs of VC2010 (upper lane) with the WB235 chromosomes (lower lane). Several small indels (<100 Kb) are apparent throughout, some of them correspond to differences in satellite length. The largest difference is due to a 241 Kb fragment of chromosome I in WB235 (coordinates 4307986-4548931) which is absent in VC2010. This sequence is found in the aligned chromosome III of VC2010. This anomaly is not found in the alignment of WB235 with LH.
